# Development and Validation of a Tool to Assess Disease-Related Knowledge in Children with Coeliac Disease

**DOI:** 10.3390/jcm15030997

**Published:** 2026-01-26

**Authors:** Sophie Hall, Kristin Kenrick, Kirsten J. Coppell, Andrew S. Day, Angharad Vernon-Roberts

**Affiliations:** 1 Department of Paediatrics and Child Health, University of Otago Christchurch, 4 Oxford Terrace, Christchurch 8011, New Zealandangharad.hurley@otago.ac.nz (A.V.-R.); 2 Department of Paediatrics, Christchurch Hospital, 2 Riccarton Avenue, Christchurch 8011, New Zealand; 3 Department of General Practice and Rural Health, Dunedin School of Medicine, Rm 124, 55 Hannover Street, Dunedin 9016, New Zealand; 4 Department of Medicine, University of Otago Wellington, P.O. Box 7343, Wellington 6242, New Zealand; kirsten.coppell@otago.ac.nz

**Keywords:** coeliac disease, knowledge, content validity, readability, validation, children

## Abstract

**Background/Objectives:** Assessment of disease-specific knowledge levels among children with coeliac disease (CD) is essential to support self-management of their condition. A suitable knowledge assessment tool has not yet been identified that is appropriate for children. The aim of this study was to develop and validate a tool for this purpose. **Materials and Methods:** Using content synthesis of available literature, a CD knowledge assessment tool (CD-Know) was developed to include items shown to be relevant to the management of CD for children. CD-Know went through development stages of expert review, two rounds of pilot/validation testing, and item response analysis. CD-Know scores were compared between participant groups using a univariate linear model. **Results:** CD-Know was developed using content synthesis and review by international CD experts. CD-Know was initially piloted among adults/children with CD (*n* = 12) and underwent the first validation study (*n* = 330 participants) among adults/children with CD, cohabitants, healthcare professionals (HCPs), and groups without CD. Based on item response analysis the tool was modified. The phases of the pilot (*n* = 7) and validation studies were repeated among refined groups (*n* = 230). The final 15-item CD-Know demonstrated an appropriate hierarchy of knowledge between testing groups. Children with CD scored lower than cohabitants of someone with CD (mean difference (MD) −3.0, SD 0.4, *p* < 0.001) and HCPs (MD −1.7, *p* = 0.009), at a similar level to adults without CD (MD 0.6, *p* = 0.88), and higher than children without CD (MD 5.8, *p* < 0.001). The CD-Know score of children with CD was positively associated with their adherence level to a gluten-free diet (R 0.30, *p* = 0.017). Test–retest reliability had a good intraclass correlation coefficient (R 0.73, *p* = 0.003). Internal consistency was good (Cronbach’s alpha 0.71). **Conclusions:** CD-Know is a validated tool to assess disease-related knowledge in children diagnosed with CD. Its potential applications include identifying areas for knowledge enhancement within the population and assessment of CD interventions.

## 1. Introduction

Coeliac disease (CD) is a chronic, immune-mediated enteropathy in response to ingestion of gluten, which occurs in genetically susceptible individuals [1]. It affects around 1 in 100 people internationally, both children and adults, with rates thought to be increasing [2,3]. Currently, the only treatment for CD is the lifelong strict avoidance of dietary gluten. For the majority of individuals, strict adherence to a gluten-free diet (GFD) induces remission with resolution of gastrointestinal and extra-intestinal symptoms, reduction in the risk of related potential complications of poorly managed CD, and optimization of growth in children [4,5,6,7].

Following diagnosis, it is generally expected that adhering to the GFD is self-managed by the person diagnosed with CD, or with help and support from their family, particularly for children with CD [8]. However, adherence to a GFD has its challenges both in the home and when eating out and can have a significant social and emotional impact on those diagnosed with CD and their families [9,10,11]. In order to effectively manage CD, it is essential that children with CD and their families have a good knowledge and understanding of their condition and the importance of adopting and maintaining a GFD [12]. Good knowledge of the GFD has been associated with better adherence in both adults and children with CD [13,14,15,16]. Furthermore, it has been repeatedly shown that poor adherence to the GFD and gluten contamination due to inadequate food preparation practices are common causes of ongoing symptoms for those diagnosed with CD [9,17]. It has been reported that among patients with non-responsive CD, defined as ongoing symptoms or recurrence of symptoms after at least six months of presumed adherence to a GFD, gluten contamination was evident in 30–51% of individuals [6,17,18,19,20,21]. Membership in CD advocacy groups is associated with improved adherence to a GFD [15,22]. These findings highlight the importance of high-quality education and support for those diagnosed with CD and their wider community. Consequently, it is necessary to be able to accurately assess knowledge of CD and the GFD to ensure adequate understanding and optimise management. The effectiveness of current and future management strategies and interventions could then be assessed to determine how well they improve CD knowledge and health outcomes.

A recent systematic review identified 25 CD knowledge assessment tools that assessed knowledge in various domains and in different groups [23]. None of the 25 tools had optimal generalisability, breadth, and validity for specific use in children [23]. Further, none of the studies assessed all of the following tool metrics that are known to be important for paediatric populations [23]—readability, brevity, content validity, and reliability—and no formal validation of the tools were undertaken. To create a robust knowledge assessment tool tailored to a target population, a thorough development, design, and validation process is essential to include stages of expert and target audience input, pilot/feasibility testing, and validation among population groups [24]. Therefore, the aims of this study were to design and develop a CD knowledge assessment tool for children with CD, to validate the tool among the target population, and to assess whether the tool could provide quantifiable data on knowledge about CD.

## 2. Materials and Methods

### 2.1. Design and Development Stages

The design and development of the CD knowledge assessment tool had four phases: development, content validation, pilot and validity testing (two rounds), and item response analysis (Figure S1).

#### 2.1.1. Phase 1: Development

None of the existing 25 CD knowledge assessment tools identified in the recent systematic review [23] reported all the necessary aspects to support their robustness and suitability for use in either clinical or research settings for population groups with CD, in particular, among children. Hence, it was essential that the tool developed met all pre-determined feasibility, validity/reliability, and generalisability criteria. All items included in the identified tools were extracted and independently reviewed by two assessors (SH, AVR) for relevance and inclusion for Phase 2. Relevance was defined as being appropriate for a patient knowledge survey, not too vague or broad, not including region-specific items (for example, national policies or region-specific foods), and being related to CD management post diagnosis. We specifically excluded items related to clinical presentation and diagnosis methods as the main focus of the tool was knowledge about dietary management among children with diagnosed CD.

#### 2.1.2. Phase 2: Content Validation

A preliminary CD knowledge assessment tool that used items from Phase 1 was developed, including items and answer options for each. For content validation it is suggested that 8–12 carefully selected experts are included in order to provide a wide range of feedback [25]. A group of experts in CD were contacted to provide qualitative and quantitative feedback on the preliminary set of items.

##### Methods and Analysis

Experts were asked to rate the relevance, clarity, and appropriateness of each proposed item using a 10-point Likert scale. Ratings were scored according to the response number [one = lowest rating and scores one, 10 = highest rating and scores 10]. Qualitative feedback was invited and provided in the form of free-text responses. Using the assigned scores, the content validity index [CVI] was calculated as a proportion of the highest possible overall score by all respondents. The CVI results and qualitative feedback were reviewed by three authors (SH, AVR, ASD), and items with a CVI score >0.78 were included in the first version of the tool tested for validity in Phase 3 [25]. All included items were assessed for readability using the Flesch–Kincaid readability test [26]. As the tool was intended to target a broad range of literacy levels, a readability level of >70, equivalent to an American school grade 6–7, was the target [27]. The tool was called CD-Know.

#### 2.1.3. Phase 3: Pilot and Validity Testing

##### Pilot Studies

CD-Know underwent two rounds of pilot testing, using the same methodology in each round. CD-Know was piloted among a small group of adults and children with CD in the first pilot, and children with CD in the second pilot. Participants were recruited from dietitian clinics at Christchurch Hospital, New Zealand (NZ). They were asked to complete an electronic form for CD-Know while being timed, and to provide the following: demographic details [age, sex] and feedback on readability, ease of use, and relevance. The responses were reviewed by two researchers (SH, AVR) and any issues addressed while maintaining the overall intention of each knowledge assessment item.

##### Validation Studies

Two validation studies of CD-Know were undertaken using the same methodology.

##### Study Design, Population, and Recruitment

The validation studies were of a prospective experimental design, with the first conducted between October 2023–December 2023 and the second between November 2024–January 2025. The first validation study included six different groups: adults and children with CD diagnosed at least 6 months previously, healthcare professionals (HCPs), cohabitants of children/adults with CD, and the lay public. The second validation study recruited four groups: children with CD diagnosed at least 6 months previously, HCPs, cohabitants of children/adults with CD, and the lay public. Recruitment methods were the same for both validation studies, and the survey was developed and distributed online using Qualtrics survey software (https://www.qualtrics.com/, accessed on 7 April 2023, Qualtrics, Provo, UT, USA).

Groups were recruited as follows, relevant to both validation studies:

- *Children and adults with established CD*: An opt-in recruitment approach was used and a ‘call for participation’ was distributed via the monthly Coeliac New Zealand (Coeliac NZ) newsletter emailed to a membership of 2225. Coeliac NZ is a national support group for those with CD in NZ.
- *Cohabitants of someone with CD*: As above, a ‘call for participation’ was also included in the monthly Coeliac NZ newsletter emailed to members.
- *Healthcare professionals (HCPs)*: Recruited face to face in clinical areas during their normal working hours in Christchurch Hospital, NZ. HCPs who had CD, or a household or close family member with CD, were excluded. All individuals approached agreed to participate.
- *Lay public [children and adults]*: Visitors or people attending out-patient appointments at Christchurch Hospital, NZ, were invited to participate. Those who had CD, or a household or close family member with CD, were excluded. All lay people approached agreed to participate.

##### Data Collected

All participants completed the following:

- Demographic data: Age, sex, and ethnicity, and for adult participants their highest level of education.
- Additional questions:
  ○ Cohabitants: How they were related to the person(s) with CD.
  ○ Participants with CD: Age at diagnosis and self-reported adherence to the GFD, measured using a 1–10 visual analogue scale.
- CD-Know.

The participants with CD were contacted via email after two weeks asking that they repeat CD-Know using an emailed link to assess tool reliability. Tool items and response options were presented in a fixed order throughout.

##### Sample Size

Sample size was calculated to ensure sufficient power for the validation analyses, in particular, for group-based comparisons of test scores, estimation of internal consistency, and test–retest reliability. For group-based comparisons, a minimum group size of approximately 15 participants provides >80% power to detect large between-group effects (effect size > 1.0) using analysis of variance. With an excess of 100 error degrees of freedom from analysis, there is greater power to explore more subtle differences amongst the other groups.

Internal consistency and item redundancy were conducted across the full validation sample, with the estimation of *n* = 140 to test these aspects over a spectrum of responses exceeding commonly recommended sample sizes utilized for such analyses. Test–retest reliability was assessed in a subset of 50 participants with coeliac disease, providing >80% power (two-tailed, α = 0.05) to detect moderate effects (≥0.5) and enabling stable estimation of reliability coefficients.

##### Analysis

Discriminant validity was assessed by comparing mean CD-Know scores between the groups using analysis of variance (ANOVA), with the hypothesized expectation that HCPs would have the highest scores, followed by adults with CD, cohabitants of people with CD, children with CD, then the lay public. For those with CD, test–retest reliability was analysed using paired *t*-tests and an intra-class correlation coefficient (ICC). Internal consistency was assessed using Cronbach’s alpha, with scores > 0.7 indicating acceptable reliability [28]. For individual items in the survey, the inter-item correlation (IIC) was measured, with scores between 0.15 and 0.5 showing good correlation and indicating that each item was measuring the same overall concept [29]. Results were considered significant at a level *p* < 0.05. Statistical analysis was performed using SPSS 29 (IBM Corp, Armonk, NY, USA).

##### Consent and Ethics

During the pilot studies, where participants were recruited face to face, all participants over the age of 16 years provided written consent, and those aged ≤ 15 years provided their assent, with parental consent to their participation being collected. All assessments for the validation studies were completed via Qualtrics survey software (Qualtrics, Provo, UT, USA). All adult participants (aged ≥ 16 years) provided online consent prior to being able to access the survey. Child participants provided online assent but could only access the surveys once their parents had also provided their online consent to their child taking part in the study. Ethical approval was granted by the University of Otago Human Ethics Committee (Health) (HD23/030).

#### 2.1.4. Phase 4: Item Response Analysis

##### Item Assessment

- Following the first validation study, the response patterns to each CD-Know item were assessed for those with CD. This enabled identification of items that were not performing efficiently and could be improved. Items were assessed using the following metrics:
- Difficulty index: Calculation of the percentage of respondents answering an item correctly. An acceptable range is considered to be 30–70%, with <30% correct answers indicating that the item was too difficult and >70% indicating it was too easy [30].
- Discrimination index: Establishes how well each item could distinguish between the highest- and lowest-scoring participants. Scores for the top and the bottom 27% of scorers are compared [31]. Acceptable scores are considered >0.29, with those items ≤ 0.29 indicating that the item cannot distinguish well between the high and low scorers [31].
- Distractor efficiency: The performance of alternative answers in multiple-choice questions/items (MCQ) are assessed by calculating the number of times (%) they were selected. Distractors are considered efficient if they are selected by >5% of respondents [30].

##### Item Reduction

Items were retained if they had acceptable scores for all three metrics, re-written if scores were borderline, or removed if scores indicated they were too easy/difficult or MCQs contained inefficient distractors. The edited CD-Know was retested using the same pilot and validation methodology as described above (Table S2).

## 3. Results

### 3.1. Phase 1: Tool Design and Development

The survey tools (*n* = 25) identified in a previous systematic review were retrieved and 265 individual items extracted and independently reviewed [23]. A total of 233 items were excluded for the following reasons: similar/duplicated items (*n* = 137; 59%), diagnostic items (*n* = 19; 8%), specific foods (*n* = 35; 15%), region-specific items (*n* = 11; 5%), very generalised (*n* = 24; 10%), or free text items (*n* = 7; 3%). The remaining 32 items were critically reviewed and edited by the research team. Seven items were then deleted, and two items were amalgamated with another item where the question was similar. Decisions to delete or amalgamate items were made by all members of the research team through consensus, using the same structured review process applied in earlier stages. This later refinement step focused on concept overlap, clarity, and reducing redundancy while preserving the inclusion of key knowledge domains, rather than removal of clearly duplicated or unsuitable items. The initial version of the assessment tool, now called CD-Know, contained 23 items.

### 3.2. Phase 2: Content Validation

The CD-Know tool was sent to 15 experts in CD management from Australasia, Asia, North America, and Europe. The content validity process was completed by 11 experts, including dietitians, gastroenterologists, and researchers in both adult and paediatric CD care from Australasia, Europe, and North America (Figure S2).

#### Analysis and Initial Version of CD-Know

The CVI scores were calculated for each CD-Know item. CVI scores and comments for each item were reviewed by the authors (Table S1). Of the 23 items, two items did not achieve a CVI score ≥ 0.78 in all areas and were excluded. Four items scored below 0.78 for clarity, and three scored below 0.78 for appropriateness. However, all seven items were rated 0.78 or higher for relevance, so the expert panel feedback was carefully reviewed. A further three items were removed, and changes were made to improve clarity of the other four. The readability of the remaining 18 items had a Flesch–Kincaid readability score of 68.3, slightly below the intended reading level of 70 [30].

The first 18-item version of CD-Know was a mix of five dichotomous (yes/no or true/false) items and 13 multiple-choice items/questions (MCQs), all with an answer option of ‘don’t know’. The tool was scored by assigning one point for each correct answer, with a total possible score of 20 points, as 16 items had only one correct answer and two items included two possible correct answers.

### 3.3. Phase 3: Initial Pilot and Validation Testing

Initially, CD-Know was intended to be appropriate for both children and adults with CD. As such, the first pilot study included a group of 12 people with CD (6 adults, and 6 children aged eight years and over), of whom 8 were female and aged between eight and 64 years. The 18-item CD-Know tool took a mean of 2 min and 53 s (standard deviation (SD) 36 s) for adults to complete and a mean of 4 min and 04 s (SD 70 s) for children to complete. The six adult participants scored a mean CD-Know of 18.7 (SD 0.94), equivalent to 93% (SD 4.7). The six children scored a mean of 15.8 (SD 3.3), equivalent to 79% (SD 16.7%). All participants agreed or strongly agreed that the number of items was ‘fine’, the item answer options were easily understood, and the tool was relevant and a good way to assess CD knowledge. The 18-item CD-Know was not altered further after this pilot test.

#### First Validation Study

A total of 338 people participated in the first validation study (Table 1). Given recruitment was through open invitation, it was not possible to determine the eligible population and hence the response rate. In addition, some participants did not complete the survey, but this number was not recorded.

**Figure 1:**
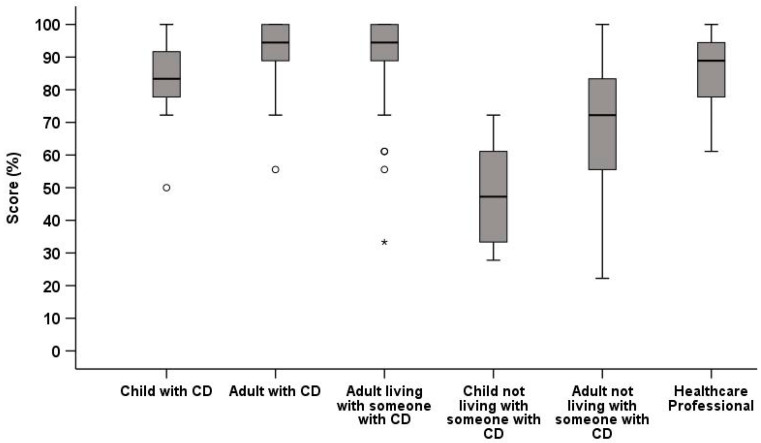
Boxplot of CD-Know scores for six participant groups in the first validation study (*n* = 338). CD = coeliac disease; * = outlier.

The mean percentage CD-Know score for each of the six groups was as follows: adults with CD, 94% (SD 7); children with CD, 84% (SD 13.4); cohabitants, 90% (SD 11); HCPs, 86% (SD 11); adults without CD, 68% (SD 23); and children without CD, 48% (SD 15). Between-group analysis showed that these scores did not differ between the groups (*p* > 0.05 for all) (Figure 1), indicating the 18-item CD-Know was unable to discriminate between groups with expected hierarchical scores. The Cronbach’s alpha was 0.69 for children and 0.53 for adults with CD, both <0.7, indicating poor internal consistency for both groups.

### 3.4. Phase 4: Item Response Analysis

#### 3.4.1. Item Response Analysis

Due to the lack of discrimination between groups in Phase 3, an item response analysis was undertaken using the answers from the children and adults with CD to each of the 18 CD-Know items (Table 2). The difficulty index was >0.7 for all items for adults and for 15 items for children, indicating that the majority were too easy for both adults and children with CD. A discrimination index of ≤0.29 was found in all but four items for adults and seven for children, showing poor discrimination between high- and low-scoring respondents. Two of the 11 MCQs showed a distractor efficiency of >5% for adults. The number of children with CD participating was insufficient to assess distractor efficiency in this group. The overall Flesch–Kincaid readability score was 68.3, with five items scoring 60–70 and six scoring < 60.

#### 3.4.2. Tool Modification

Items shown to have low difficulty and a poor discrimination index were reviewed and then modified to increase the complexity while maintaining the core focus of each item. Two items that were deemed too simple despite modifications were removed from the second iteration of the tool, leaving 16 items in the revised version of CD-Know. The target population of CD-Know was modified to be for children only with CD.

### 3.5. Phase 3 Repeated: Second Pilot and Validation Studies

#### 3.5.1. Second Pilot Study

Pilot testing of the revised version of CD-Know was undertaken among a group of seven children with CD aged between 8–17 years, of whom four were female. The 16-item CD-Know took a mean of 3 min and 5 s (SD 49 s) to complete. The mean score achieved was 10.3 (SD 3.6), equivalent to 64% (SD 22). Children agreed/strongly agreed that the number of items was ‘fine’, the answer options were easily understood, and the tool was a good measure of CD knowledge. However, two children somewhat disagreed that the items were easily understood. On review of the second pilot results, one item answered correctly by all children was removed, and the wording of another simplified. The revised version of CD-Know consisted of 15 items: nine MCQ and six true/false or yes/no items, with all items having only one correct answer, meaning a maximum score of 15 (Appendix A).

#### 3.5.2. Second Validation Study

**Figure 2:**
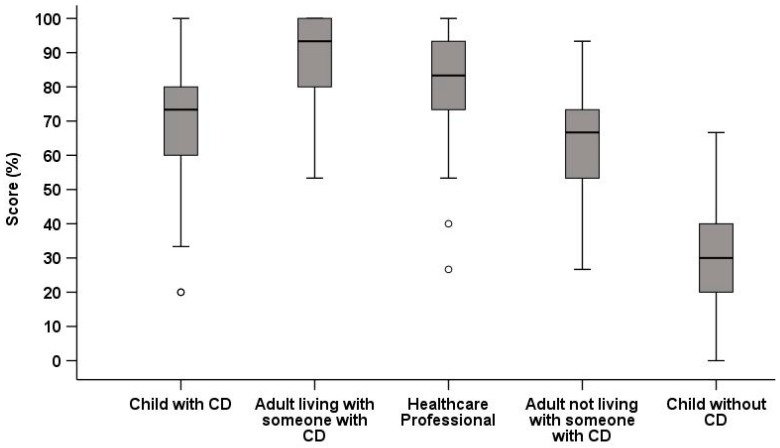
Boxplot of CD-Know scores for the five participant groups in the second validation study (*n* = 230). CD = coeliac disease.

A total of 230 people participated in the second validation study (Table 3). Children with CD scored lower than the cohabitants of someone with CD (MD −3.0, SD 0.4, *p* < 0.001) and HCPs (MD −1.7, *p* = 0.009), at a similar level to adults that did not have CD (MD 0.6, *p* = 0.88), and higher than children without CD (MD 5.8, *p* < 0.001) (Figure 2). For the group of children with CD, there was no difference between males and females (MD −0.5, *p* = 0.95) or between the different age groups (*p* > 0.05). There was a positive correlation between self-reported GFD adherence level and CD-Know scores for children with CD (R 0.30, *p* = 0.017), who had scored their adherence to the GFD as a mean of 9.7 (6.6) from a maximum of 10. The Cronbach’s alpha was 0.71 for children with CD and 0.82 for the total group, indicating acceptable internal consistency with the analysis, showing that this value would not be improved if any individual item was removed. For the total group, the IIC for each item compared to all others was within the acceptable range. Twenty children repeated CD-Know approximately two weeks later (mean of 16 days, SD 4), and their scores showed good correlation between the baseline and repeat completion (R 0.73, *p* = 0.003).

#### 3.5.3. Percentage Answering Each Item Correctly

**Figure 3:**
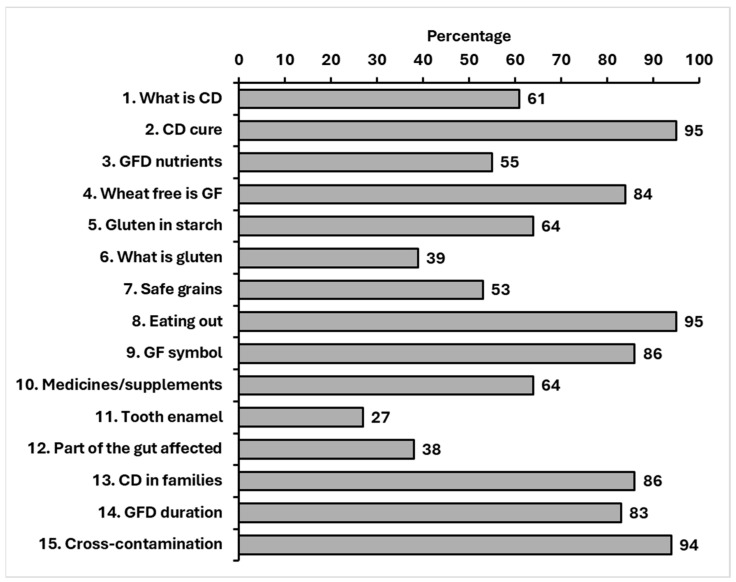
Percentage of correctly answered items in the 15-item CD-Know among children with CD in the second validation study (*n* = 64). CD = coeliac disease; GF = gluten free; GFD = gluten-free diet.

The three items answered correctly with the lowest frequency were ‘What is gluten?’, ‘Which part of the gut is affected by coeliac disease?’, and ‘Coeliac disease may cause tooth enamel problems” (Figure 3). Other items with ≥30% don’t know responses looked at identifying gluten in medicines and food products, safe grains to eat, and the effect of a GFD on nutritional intake.

In order to assess whether readability of CD-Know items may have influenced the response rate between the age groups of children with CD, the percentage of participants getting each item correct in each age group was calculated. This showed that there was no difference between the percentage with correct responses for any item (*p* > 0.05), with the exception of item number nine that had a Flesch–Kincaid readability score of 61.3. Item number nine was scored correctly by 24 (75%) children aged 8–11 years, 19 (100%) children aged 12–15 years, and 13 (100%) children aged 16–17 years (X^2^ 6.7, *p* = 0.04).

## 4. Discussion

The development of the 15-item CD-Know tool included rigorous phases of content validation, pilot testing, item response analysis, and validity and reliability assessments. The final 15-item CD-Know tool was refined through an iterative process to ensure it was relevant, readable, and repeatable for the target population of children with CD. The final version established an appropriate hierarchy of scores among participant groups and had good internal consistency, supporting its use as a paediatric disease-related knowledge assessment tool.

The development of self-management skills for children with chronic conditions begins in early childhood, and appropriate support throughout this process can facilitate improvements in well-being and health outcomes [32,33]. Knowledge is a fundamental component of self-management, enabling individuals to make informed choices in what may be, for children with CD, varied and often unpredictable food environments [34]. Several studies have identified a link between knowledge of a GFD and improved adherence to the GFD, showing improving quality of life and reducing associated symptoms [13,14]. In the current study, a correlation was seen between higher CD-Know scores and greater self-reported GFD adherence among children, as seen in previous work [12,16,35]. However, adherence was measured using a self-reported visual analogue scale and, hence, should be interpreted cautiously. A meta-analysis by Riberio et al. [36] demonstrated strong correlations between self-reported adherence and serological markers, with higher results only achieved with the use of interviews, which would not have been feasible in the current study.

Similar associations have been reported in other chronic conditions, where greater self-management knowledge in children correlates with improved adherence to prescribed treatment regimens [37]. This is particularly relevant for children with CD as they gradually develop increasing independence in managing their diet, particularly in settings where direct parental supervision is limited, such as in school, social gatherings, and extracurricular activities. Although several studies have evaluated CD-related knowledge among parents [16,38,39,40], relatively few have assessed CD knowledge in children themselves, despite their growing autonomy in making food-related choices [41,42,43].

In the current study, the paediatric cohort with CD had limited understanding of the definition of gluten, the site of gastrointestinal involvement in CD, and awareness of extraintestinal signs such as dental enamel defects. Additionally, nearly one-third of children (~30%) selected ‘don’t know’ when asked to identify gluten-containing foods, grains, and medications/supplements. This aligns with a study by Pohoreski et al. [42], who found only 37% of adolescents demonstrated ‘sufficient knowledge’ of the GFD, particularly when identifying gluten-free foods. While Pohoreski et al. [42] primarily assessed recognition of GF foods, CD-Know extends this approach by incorporating disease awareness, decision-making and cross-contamination scenarios, which may better reflect the real-world self-management challenges faced by children. Insufficient knowledge about gluten-containing foods is likely to increase the risk of inadvertent gluten exposure among children, particularly outside the family home, thereby contributing to ongoing or persistent symptoms or associated health issues/adverse outcomes [9].

A number of educational strategies for children with CD have already been developed to support increasing knowledge levels. These include e-Learning modules, picture books, video conferencing, and in-person group and individual dietitian-led sessions [44,45,46,47,48]. A validated knowledge assessment tool could be a valuable addition in the assessment of pre- and post-intervention measures as well as assessment of long-term knowledge retention to objectively measure the effectiveness of these strategies. Importantly, CD-Know is intended to complement, rather than replace, clinical assessment and education, and should not be interpreted as a proxy measure of disease control or dietary adherence.

Wider validation of CD-Know is also warranted in other populations such as the food service industry, where gaps in knowledge have previously been identified, in particular regarding ensuring the safety of meals offered as “gluten free” [49,50]. A study by Khafagy et al. [50] found that only 17.5% of chefs and restaurant owners in two cities in western Saudi Arabia had heard of CD, with only 34.1% able to identify gluten-containing items. An NZ study found that although 87% of staff in food outlets offering GF options had heard of CD, only 24.4% were aware of necessary GF food preparation policies [51]. In a Canadian hospital food service setting, Zhou et al. [49] found that frontline staff scored lower than supervisors (71.7% vs. 87.9%) when GF knowledge was assessed, with only 36% having had formal training on the GFD. Unlike the current study, these studies focused on knowledge among food handlers rather than individuals with CD, and therefore prioritized food handling, cross-contamination, and ingredient identification rather than holistic knowledge of CD. However, their findings underline the significant influence of food service knowledge on the safety and feasibility of eating outside the home for people with CD. A knowledge assessment tool that assesses general knowledge of CD, beyond the ability to identify gluten-containing food items, such as CD-Know, may offer valuable insights into which specific aspects of CD and its management are lacking in these groups. Promoting a comprehensive understanding of CD as a chronic medical condition may foster a greater awareness that dietary restrictions for those with CD are medically necessary and even trace amounts of gluten can have significant health implications.

An important consideration in interpreting CD-Know relates to how ‘knowledge’ is defined. The final instrument prioritizes knowledge that supports everyday self-management in food-related contexts but also includes understanding the immune-mediated and lifelong nature of CD, organ involvement, and familial risk. While this extends beyond purely a food recognition checklist, the emphasis remains on practical knowledge required to reduce inadvertent gluten exposure in everyday environments. CD-Know does not assess awareness of severe and atypical manifestations of CD. Limited knowledge of these may have important clinical consequences, including delayed recognition of serious complications. Although inclusion of such content may not be developmentally appropriate for younger children, this narrowing represents a deliberate boundary of the current tool and should be explicitly acknowledged. Future research could support the development of age-stratified extensions that incorporate higher-risk clinical knowledge relevant to older adolescents and young adults.

Randomization of item order was deliberately not carried out in this study, as several questions risked introducing confusion or inadvertently alluding to answers to subsequent questions. No feedback on correct responses was provided between administrations, which may have limited the potential learning effects of this. Nevertheless, we acknowledge this as a limitation of the study. Readability was carefully considered during tool development, with an overall target aligned to American school grade 6–7 reading levels. However, several individual items did not meet the prespecified readability threshold, which may disproportionately affect younger children or those with lower literacy levels. Although no statistically significant differences in total CD-Know score, nor for scores for items that exceeded the readability threshold, were observed between predefined age groups, age was not assessed as a continuous predictor of score or item comprehension. Future validation studies should include analyses examining the relationship between age, literacy, “don’t know” responses, and overall performance to further support suitability across the full 8–17 years age range.

The target population of children with CD was recruited through the consumer group Coeliac NZ on an opt-in basis, which likely selected for participants who were more engaged and knowledgeable group than those who were not members of Coeliac NZ. This may have inflated baseline knowledge scores and reduced variability, limiting the generalisability of findings to less engaged populations, including those not affiliated with advocacy organizations or groups with lower health literacy. Although the study endeavoured to ensure that CD-Know is applicable to other populations and groups, it requires further validation studies in other countries to ensure reproducibility of results, thereby establishing generalisability.

Despite these limitations, CD-Know went through a robust, transparent, and iterative process of design, development, and validation. The inclusion of international experts in CD during development, and the inclusion of diverse participant groups during validation, helped ensure that the tool was both relevant and broadly applicable to the NZ population and beyond. CD-Know items are based on internationally recognized knowledge domains relevant to the management of CD, thereby optimizing generalisability as it avoids region-specific content related to local foods, legislation, or healthcare systems.

## 5. Conclusions

CD-Know addresses key limitations of existing CD knowledge assessment tools, which have typically been designed for adults, lacked validation, or failed to comprehensively assess the domains critical to effective disease self-management. CD-Know provides a quick, standardized method to quantify CD knowledge and identify areas for knowledge enhancement that could be practically applied to enable tailored patient care. CD-Know may also be implemented during the development and testing of future CD educational interventions and has further potential research applications to improve clinical outcomes. Future research should explore the generalisability of CD-Know across different populations and healthcare settings, as well as other non-clinical areas such as the food service industry. Ultimately, CD-Know has the potential to enhance patient education, support individualized care, and contribute to improved long-term outcomes for children living with CD.

## Figures and Tables

**Table 1:** Demographic description of the six groups participating in the first validation study (*n* = 338).

		Children with CD *n* (%)	Adults with CD *n* (%)	Cohabitants *n* (%)	HCP *n* (%)	Adults Without CD *n* (%)	Children Without CD *n* (%)
	Sample Size	15	183	81	28	17	14
Gender	Male	6 (40)	15 (8)	8 (10)	3 (11)	5 (29)	5 (36)
	Female	9 (60)	165 (90)	73 (90)	25 (89)	12 (71)	8 (57)
	Non-binary	0	2 (1)	0	0	0	1 (7)
	Prefer not to say	0	1 (1)	0	0	0	0
Age	Children 8–11	10 (67)	0	10 (12)	0	0	6 (43)
	Children 12–14	1 (7)	0	6 (7)	0	0	4 (28.5)
	Children 15–17	4 (26)	0	4 (5)	0	0	4 (28.5)
	Young adult (18–34)	0	34 (18)	8 (10)	16 (57)	2 (12)	0
	Middle age (35–54)	0	71 (39)	48 (59)	10 (36)	10 (59)	0
	Mature (55+)	0	78 (43)	5 (6)	2 (7)	5 (29)	0
Ethnicity *	NZ European	14 (93)	168 (92)	74 (91)	25 (89)	15 (88)	14 (100)
	Māori	1 (7)	6 (3)	5 (6)	0	0	3 (21)
	Pacific Island	0	1 (0.5)	0	0	3 (18)	3 (21)
	Asian	0	2 (1)	0	0	2 (12)	0
	MELAA	1 (7)	1 (0.5)	0	0	0	0
	Other	0	17 (9)	8 (10)	3 (11)	0	1 (7)
Education	High school		28 (15)	9 (11)	0	3 (18)	
	Polytechnic/vocational training		42 (23)	9 (11)	1 (3.5)	4 (23.5)	
	University		63 (34)	32 (40)	11 (39)	5 (29)	
	Postgraduate		49 (27)	25 (31)	15 (53.5)	4 (23.5)	
	Prefer not to say		1 (1)	6 (7)	1 (4)	1 (6)	
HCP	Medical				10 (36)		
	Nursing/midwifery				1 (3)		
	Dietitian				9 (32)		
	Other allied health				3 (11)		
	Not specified				5 (18)		

* More than one option could be selected. MELAA = Middle Eastern, Latin American, or African; HCP = healthcare professional; CD = coeliac disease; *n* = number; NZ = New Zealand.

**Table 2:** Item response analysis results for the CD-Know responses by adults and children with CD in the first validation study.

Item		Format	Difficulty		Discrimination		Distractor Efficiency %	Readability
#	Text		Child	Adult	Child	Adult	Adult	
1	Which one of these describes coeliac disease?	MCQ	0.8	0.9	0.5	0.11	All ≤ 2	64.4
2	Can coeliac disease be cured?	T/F	0.9	1.0	0.25	0.04	-	66.4
3	When might people find out they have coeliac disease?	MCQ	0.9	1.0	0.25	0.04	All ≤ 1	79.3
4	How is coeliac disease currently treated?	MCQ	0.9	1.0	0.25	0.0	All 0	31.5
5	What diet should people with coeliac disease follow?	MCQ	1.0	1.0	0.00	0.0	All 0	46.4
6	A gluten-free diet may be low in:	MCQ	0.4	0.8	0.75	0.39	All ≤ 3	88.7
7	What is gluten?	MCQ	0.5	0.9	0.75	0.37	6%, 4%	93.8
8	Wheat free is the same as gluten free.	T/F	0.9	1.0	0.25	0.04	-	100
9	Which grains can be safely eaten by people with coeliac disease?	MCQ	0.7	0.8	0.75	0.5	All ≤ 3	72.3
10	Some naturally gluten-free foods may have gluten added to them.	T/F	0.9	0.9	0.25	0.2	-	68.8
11	Which statement about medicines is true	MCQ	1.0	0.9	0.00	0.09	All ≤ 1	56.9
12	If a restaurant menu states a dish may contain gluten, what should someone with coeliac disease do?	MCQ	1.0	0.9	0.00	0.11	All ≤ 4	54.2
13	What kitchen items are NOT safe for people with coeliac disease to share with people who eat gluten?	MCQ	0.8	0.8	0.00	0.28	6%, 1%	69.8
14	How strict should the diet be for someone with coeliac disease?	MCQ	1.0	1.0	0.00	0.02	All ≤ 1	46.8
15	How do you decide if a packet of potato chips contains gluten?	MCQ	1.0	1.0	0.00	0.0	All 0	67.8
16	What does the international symbol for gluten free look like?	MCQ	0.9	1.0	0.50	0.02	All ≤ 1	61.3
17	All herbal medicines and supplements are gluten free.	T/F	0.5	0.8	0.50	0.41	-	53.9
18	If a food label says ‘starch’, it always contains gluten?	T/F	0.8	0.9	0.50	0.22	-	78.2

MCQ—multiple-choice question; T/F—true/false item; - = not applicable.

**Table 3:** Participant demographics for the second validation study, including additional details for cohabitants and healthcare professional groups (*n* = 230).

		Children with CD *n* (%)	Cohabitants *n* (%)	HCP *n* (%)	Adults Without CD *n* (%)	Children Without CD *n* (%)
*n*	Sample Size	64	96	36	20	14
Gender	Male	20 (31)	8 (8)	8 (22)	7 (35)	7 (50)
	Female	44 (69)	87 (91)	27 (75)	13 (65)	7 (50)
	Prefer not to say	0	1 (1)	1 (3)	0	0
Age	Children 8–11	32 (50)	0	0	0	6 (43)
	Children 12–14	19 (30)	0	0	0	6 (43)
	Children 15–17	13 (20)	0	0	0	2 (14)
	Young adult (18–34)	0	4 (4)	15 (42)	10 (50)	0
	Middle age (35–54)	0	88 (92)	17 (47)	10 (50)	0
	Mature (55+)	0	4 (4)	4 (11)	0	0
Ethnicity *	NZ European	62 (97)	85 (89)	27 (75)	15 (75)	14 (100)
	Māori	6 (9)	5 (5)	2 (6)	0	0
	Pacific Island	1 (2)	0	0	1 (5)	4 (29)
	Asian	2 (3)	0	2 (6)	3 (15)	0
	MELAA	1 (2)	1 (1)	0	0	0
	Other	1 (2)	11 (11)	8 (22)	3 (15)	0
	Prefer not to say	0	1 (1)	1 (3)	0	0
Education	High school		14 (15)	0	5 (25)	
	Polytechnic/vocational training		21 (22)	1 (3)	1 (5)	
	University		33 (34)	14 (39)	7 (35)	
	Postgraduate		26 (27)	21 (58)	7 (35)	
	Prefer not to say		2 (2)	0	0	
HCP	Medical			11 (31)		
	Nursing/midwifery			15 (42)		
	Dietitian			7 (19)		
	Other allied health			3 (8)		

* More than one option could be selected. NZ = New Zealand; MELAA = Middle Eastern, Latin American, or African; HCP = healthcare professional; CD = coeliac disease; *n* = number.

## Data Availability

The original contributions presented in this study are included in the article/Supplementary Materials. Further inquiries can be directed to the corresponding author.

## Supplementary Materials

The following supporting information can be downloaded at: https://www.mdpi.com/article/10.3390/jcm15030997/s1, Figure S1: Study flow chart to explain phases of the research, with repeated items; Figure S2: Professional details of Expert Panel members (*n* = 11) and their clinical experience in caring for adults or children with CD; Table S1: Results of the Content Validity Index (CVI) assessment (relevance, clarity, appropriateness) for each item in the initial 23-item CD-Know version and whether each item was retained, removed or modified. Table S2: Original versus revalidated CD-Know tool.

## Appendix A

**Table:**

	CD-Know
**1**	Which of these best describes coeliac disease? (a) An enzyme deficiency **(b)** **An immune response** (c) An allergic response (d) Don’t know
**2**	Which statement about coeliac disease is true? (a) You can grow out of it **(b)** **It is lifelong** (c) Medications can control symptoms (d) Don’t know
**3**	A gluten-free diet may be low in: **(a)** **Fibre** (b) Fat (c) Calories (d) Don’t know
**4**	If a food states it is wheat free, then it is always gluten free (a) True **(b)** **False** (c) Don’t know
**5**	If a food label says ‘starch’, it always contains gluten. (a) True **(b)** **False** (c) Don’t know
**6**	What is gluten? **(a)** **A protein** (b) A fat (c) A carbohydrate (d) Don’t know
**7**	Which grains can be safely eaten by people with coeliac disease? (select one option) (a) Buckwheat, rye, semolina **(b)** **Polenta, quinoa, glutinous rice** (c) Glutinous rice, cous-cous, buckwheat (d) Don’t know
**8**	You order a gluten-free burger but it arrives with a regular bun. What should you do? **(a)** **Ask for a new burger to be made** (b) Ask to have the patty put in a gluten-free bun (c) Just eat the patty (d) Don’t know
**9**	What does the international symbol for ‘gluten free’ look like? **(a)** **A crossed grain** (b) A crossed slice of bread (c) A gluten traffic light (d) Don’t know
**10**	All herbal medicines and supplements are gluten free. (a) True **(b)** **False** (c) Don’t know
**11**	Coeliac disease may cause tooth enamel problems **(a)** **True** (b) False (c) Don’t know
**12**	Which part of the gut is affected by coeliac disease? (a) Stomach **(b)** **Small bowel** (c) Large bowel (d) Don’t know
**13**	If I have coeliac disease, my parents and siblings are: **(a)** **More likely to have coeliac disease** (b) Less likely to have coeliac disease (c) The same as everyone else (d) Don’t know
**14**	Can someone with coeliac disease have gluten 6 months after diagnosis if they have no symptoms? (a) Yes **(b)** **No** (c) Don’t know
**15**	Gluten-free food cooked on a barbecue/grill is always safe: (a) Yes **(b)** **No** (c) Don’t know

Correct answer in bold. Each correct answer scored as one point to a maximum of 15.

## References

[1] Fasano A., Catassi C. (2012). Clinical practice: Celiac disease. N. Engl. J. Med..

[2] King J.A., Jeong J., Underwood F.E., Quan J., Panaccione N., Windsor J.W., Coward S., deBruyn J., Ronksley P.E., Shaheen A.A. (2020). Incidence of Celiac Disease Is Increasing Over Time: A Systematic Review and Meta-analysis. Am. J. Gastroenterol..

[3] Roberts S.E., Morrison-Rees S., Thapar N., Benninga M.A., Borrelli O., Broekaert I., Dolinsek J., Martin-de-Carpi J., Mas E., Miele E. (2021). Systematic review and meta-analysis: The incidence and prevalence of paediatric coeliac disease across Europe. Aliment. Pharmacol. Ther..

[4] Troncone R., Kosova R. (2010). Short Stature and Catch-up Growth in Celiac Disease. J. Pediatr. Gastroenterol. Nutr..

[5] Barratt S.M., Leeds J.S., Sanders D.S. (2013). Factors influencing the type, timing and severity of symptomatic responses to dietary gluten in patients with biopsy-proven coeliac disease. J. Gastrointest. Liver Dis..

[6] Leffler D.A., Dennis M., Hyett B., Kelly E., Schuppan D., Kelly C.P. (2007). Etiologies and Predictors of Diagnosis in Nonresponsive Celiac Disease. Clin. Gastroenterol. Hepatol..

[7] Valdimarsson T., Löfman O., Toss G., Ström M. (1996). Reversal of osteopenia with diet in adult coeliac disease. Gut.

[8] Mearin M.L., Agardh D., Antunes H., Al-toma A., Auricchio R., Castillejo G., Catassi C., Ciacci C., Discepolo V., Dolinesk J. (2022). ESPGHAN Position Paper on Management and Follow-up of Children and Adolescents With Celiac Disease. J. Pediatr. Gastroenterol. Nutr..

[9] Coppell K.J., Stamm R.A., Sharp K.P.H. (2019). Diagnostic delays and treatment challenges in children with coeliac disease: The New Zealand Coeliac Health Survey. N. Z. Med. J..

[10] Russo C., Wolf R.L., Leichter H.J., Lee A.R., Reilly N.R., Zybert P., Green P., Lebwohl B. (2020). Impact of a Child’s Celiac Disease Diagnosis and Management on the Family. Dig. Dis. Sci..

[11] Rozensztrauch A., Mostyńska P. (2025). Quality of Life in Children with Celiac Disease: An Observational Study. Nutrients.

[12] Elsahoryi N.A., Altamimi E., Subih H.S., Hammad F.J., Woodside J.V. (2020). Educational Intervention Improved Parental Knowledge, Attitudes, and Practices (KAP) and Adherence of Patients with Celiac Disease to Gluten-Free Diet. Int. J. Food Sci..

[13] Leffler D.A., Edwards-George J., Dennis M., Schuppan D., Cook F., Franko D.L., Blom-Hoffman J., Kelly C. (2008). Factors that Influence Adherence to a Gluten-Free Diet in Adults with Celiac Disease. Dig. Dis. Sci..

[14] Halmos E.P., Deng M., Knowles S.R., Sainsbury K., Mullan B., Tye-Din J.A. (2018). Food knowledge and psychological state predict adherence to a gluten-free diet in a survey of 5310 Australians and New Zealanders with coeliac disease. Aliment. Pharmacol. Ther..

[15] Abu-Janb N., Jaana M. (2020). Facilitators and barriers to adherence to gluten-free diet among adults with celiac disease: A systematic review. J. Hum. Nutr. Diet..

[16] Charalampopoulos D., Panayiotou J., Chouliaras G., Zellos A., Kyritsi E., Roma E. (2013). Determinants of adherence to gluten-free diet in Greek children with coeliac disease: A cross-sectional study. Eur. J. Clin. Nutr..

[17] Malamut G., Soderquist C.R., Bhagat G., Cerf-Bensussan N. (2024). Advances in Nonresponsive and Refractory Celiac Disease. Gastroenterology.

[18] Abdulkarim A.S., Burgart L.J., See J., Murray J.A. (2002). Etiology of nonresponsive celiac disease: Results of a systematic approach. Am. J. Gastroenterol..

[19] Dewar D.H. (2012). Celiac disease: Management of persistent symptoms in patients on a gluten-free diet. World J. Gastroenterol..

[20] Veeraraghavan G., Therrien A., Degroote M., McKeown A., Mitchell P.D., Silvester J.A., Leffler D., Leichtner A., Kelly C., Weir D. (2021). Non-responsive celiac disease in children on a gluten free diet. World J. Gastroenterol..

[21] Aggarwal N., Bhatia U., Dwarakanathan V., Singh A.D., Singh P., Ahuja V., Makharia G.K. (2025). Prevalence and etiologies of non-responsive celiac disease: A systematic review and meta-analysis. J. Gastroenterol. Hepatol..

[22] Hall N.J., Rubin G., Charnock A. (2009). Systematic review: Adherence to a gluten-free diet in adult patients with coeliac disease. Aliment. Pharmacol. Ther..

[23] Hall S., Kenrick K., Day A.S., Vernon-Roberts A. (2024). A Systematic Review of Tools to Assess Coeliac Disease-Related Knowledge. J. Clin. Med..

[24] Lynn M.R. (1986). Determination and Quantification Of Content Validity. Nurs. Res..

[25] Polit D., Beck C., Owen S. (2007). Is the CVI an acceptable indicator of content validity? Appraisal and recommendations. Res. Nurs. Health.

[26] Flesch R. (1962). The Art of Plain Talk.

[27] Doak L., Doak C. (2010). Writing for readers with a wide range of reading skills. AMWA J..

[28] Litwin M.S. (1995). How to Measure Survey Reliability and Validity.

[29] Clark L.A., Watson D. (1995). Constructing Validity: Basic Issues in Objective Scale Development. Psychol. Assess..

[30] Date A.P., Borkar A.S., Badwaik R.T., Siddiqui R.A., Shende T.R., Dashputra A.V. (2019). Item analysis as tool to validate multiple choice question bank in pharmacology. Int. J. Basic Clin. Pharmacol..

[31] McGahee T.W., Ball J. (2009). How to Read and Really Use an Item Analysis. Nurs. Educ..

[32] Saxby N., Ford K., Beggs S., Battersby M., Lawn S. (2020). Developmentally appropriate supported self-management for children and young people with chronic conditions: A consensus. Patient Educ. Couns..

[33] Bravo L., Killela M.K., Reyes B.L., Santos K.M.B., Torres V., Huang C.-C., Jacob E. (2020). Self-Management, Self-Efficacy, and Health-Related Quality of Life in Children With Chronic Illness and Medical Complexity. J. Pediatr. Health Care.

[34] Modi A.C., Pai A.L., Hommel K.A., Hood K.K., Cortina S., Hilliard M.E., Guilfoyle S., Gray W., Drotar D. (2012). Pediatric self-management: A framework for research, practice, and policy. Pediatrics.

[35] Pancheva R., Dolinsek J., Panayotova M., Yankov I., Kofinova D., Nikolova S., Baycheva M., Georgieva M. (2025). Bridging the Gap: Awareness, Knowledge, and Challenges of Living with Celiac Disease in Bulgaria. Nutrients.

[36] Ribeiro C.D.S., Uenishi R.H., Domingues A.D.S., Nakano E.Y., Botelho R.B.A., Raposo A., Zandonadi R.P. (2024). Gluten-Free Diet Adherence Tools for Individuals with Celiac Disease: A Systematic Review and Meta-Analysis of Tools Compared to Laboratory Tests. Nutrients.

[37] Lim J.K., Lee Y.J., Park J.H. (2020). Medication-Related Knowledge and Medication Adherence in Pediatric and Adolescent Patients with Inflammatory Bowel Disease. J. Korean Med. Sci..

[38] Garg A., Gupta R., Zuccotti G.V. (2014). Predictors of Compliance to Gluten-Free Diet in Children with Celiac Disease. Int. Sch. Res. Not..

[39] Tomlin J., Slater H., Muganthan T., Beattie R.M., Afzal N.A. (2015). Parental knowledge of coeliac disease. Inf. Inform. Health Soc. Care.

[40] Weisbrod V.M., Kerzner B., Coburn S.S., McMahon J.P., Raber C., Raber B.M., Parker M., Stern L., Kahn I., Sady M. (2019). Assessment of Parental Gluten-Free Diet Knowledge in a Multi-Disciplinary Celiac Disease Clinic. Gastroenterology.

[41] Vázquez-Polo M., Churruca I., Perez-Junkera G., Larretxi I., Lasa A., Esparta J., Cantero-Ruiz de Eguino L., Navarro V. (2024). Study Protocol for a Controlled Trial of Nutrition Education Intervention about Celiac Disease in Primary School: ZELIAKIDE Project. Nutrients.

[42] Pohoreski K., Horwitz S.L., Gidrewicz D. (2023). Gluten-Free Diet Knowledge and Adherence in Adolescents with Celiac Disease: A Cross-Sectional Study. JPGN Rep..

[43] Ljungman G., Myrdal U. (1993). Compliance in teenagers with coeliac disease-a Swedish follow-up study. Acta Paediatr..

[44] Connan V., Marcon M.A., Mahmud F.H., Assor E., Martincevic I., Bandsma R.H., Vresk L., Walsh C. (2019). Online education for gluten-free diet teaching: Development and usability testing of an e-learning module for children with concurrent celiac disease and type 1 diabetes. Pediatr. Diabetes.

[45] McKeon L., Gildersleeve J., Mullens A.B. (2025). The Strategies of Picture Books as a Mode of Health Communication for Young Children with Coeliac Disease. Children.

[46] Rashid M., Haskett J., Parkinson McGraw L., Noble A., van Limbergen J., Otley A. (2021). Teaching Families of Children with Celiac Disease about Gluten-Free Diet Using Distributed Education: A Pilot Study. Can. J. Diet. Pract. Res..

[47] Matschull L., Martin N., Goday P., Chugh A. (2022). Evaluation of In-Person, Gluten-Free Diet Education for Children With Celiac Disease. JPGN Rep..

[48] Suárez-González M., Bousoño García C., Jiménez Treviño S., Iglesias Cabo T., Díaz Martín J.J. (2020). Influence of nutrition education in paediatric coeliac disease: Impact of the role of the registered dietitian: A prospective, single-arm intervention study. J. Hum. Nutr. Diet..

[49] Zhou F., Mullen T., Kulai T., Rashid M. (2023). Assessment of Knowledge of Gluten-Free Diet Amongst Food Handlers in Hospitals. Can. J. Diet. Pract. Res..

[50] Khafagy A.A., Qari W.K., Filimban S.S., Bahalaq A.M., Bulkhi A.A. (2022). A Cross-Sectional Study of Celiac Disease Awareness in the Food Industry in the Western Region of Saudi Arabia. Cureus.

[51] Schultz M., Shin S., Coppell K.J. (2017). Awareness of coeliac disease among chefs and cooks depends on the level and place of training. Asia Pac. J. Clin. Nutr..

