# Supplementary material for: Development and Validation of a Tool to Assess Disease-Related Knowledge in Children with Coeliac Disease
Source: J Clin Med. 2026 Jan 26;15(3):997. doi: 10.3390/jcm15030997 (PMC12898186; doi:10.3390/jcm15030997)
Supplement: Supplementary file 1 [file jcm-15-00997-s001.zip › jcm-4066389-Table S1 and figures.pdf]

Figure S1. Study flow chart to explain phases of the research, with repeated items.

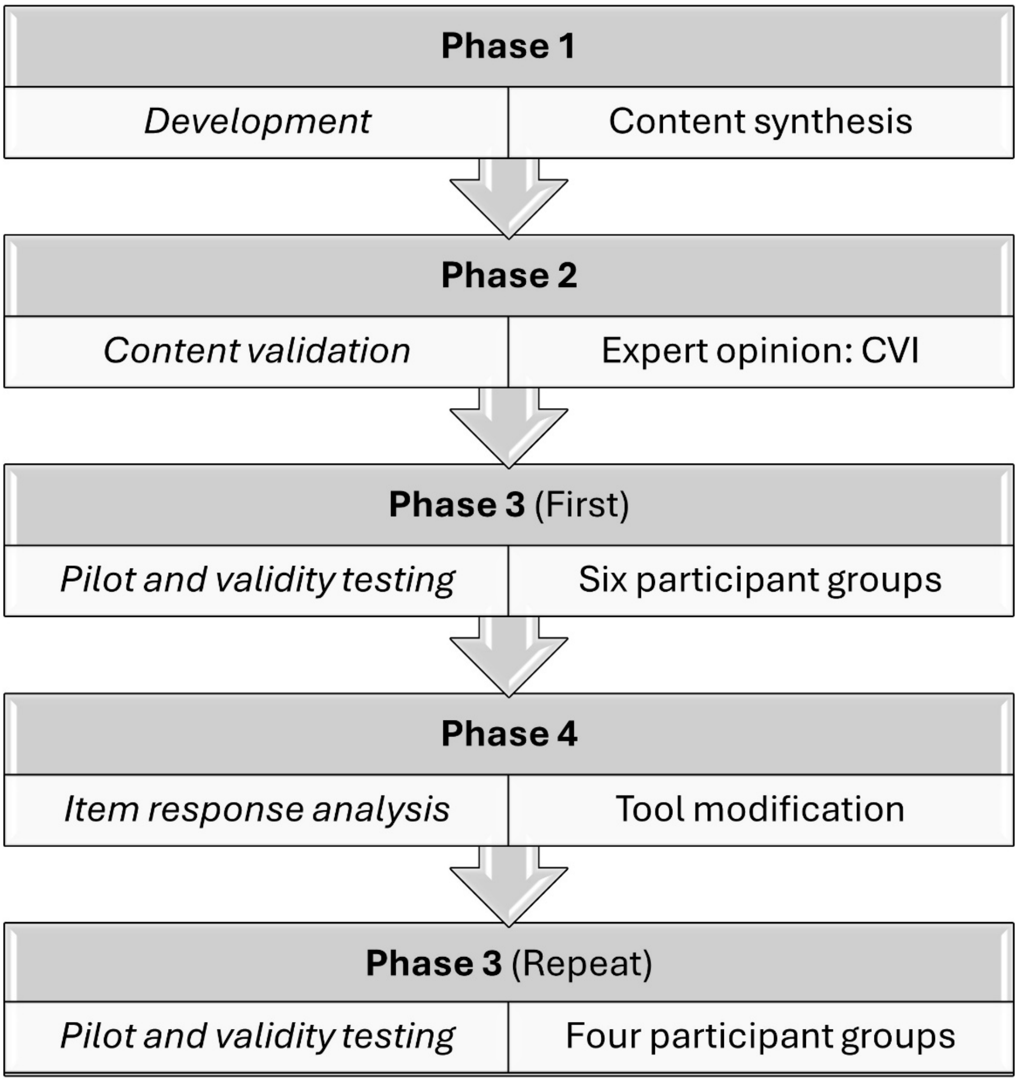

Figure S2. Professional details of Expert Panel members (n=11) and their clinical experience in caring for adults or children with CD

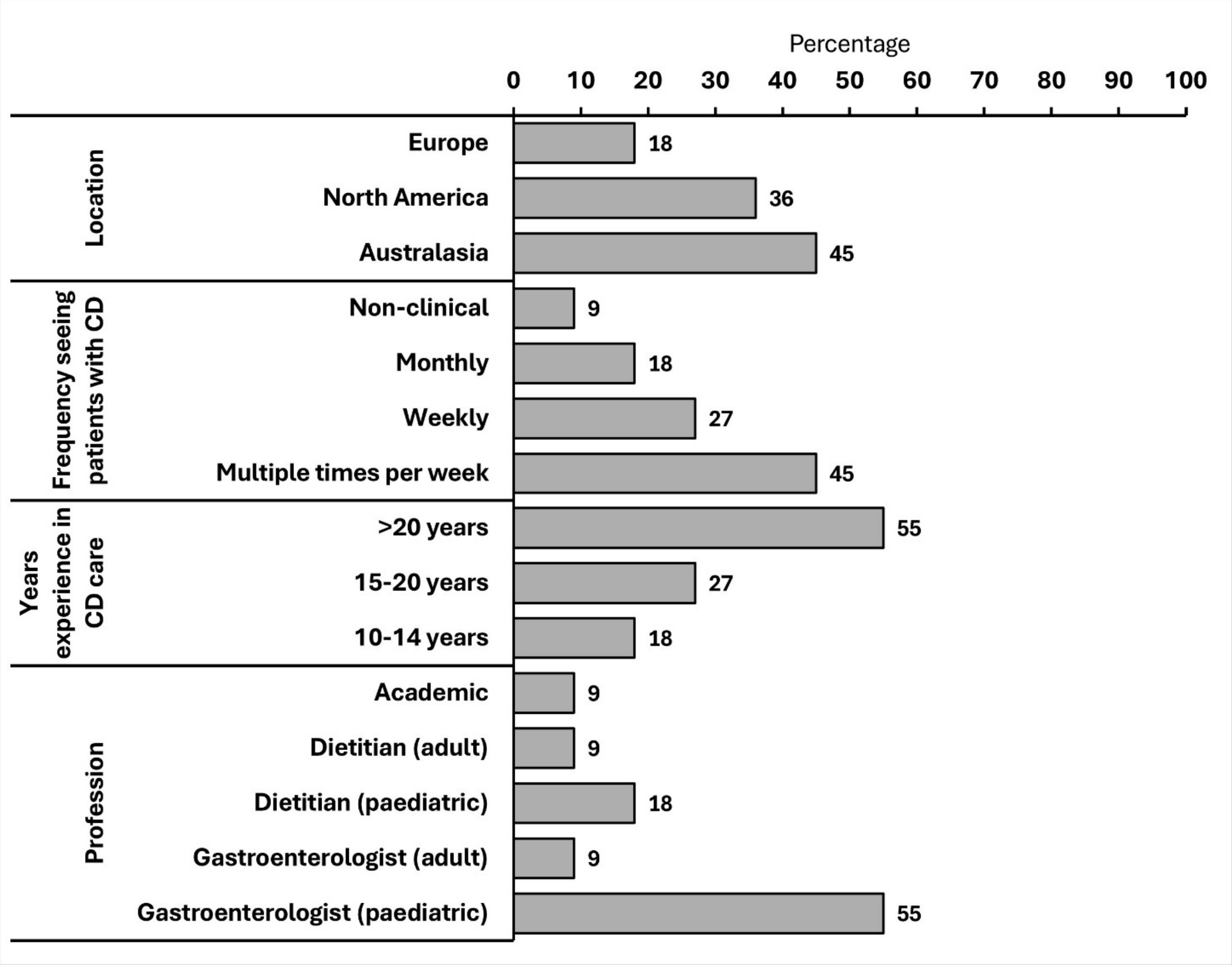

CD = Coeliac disease

Table S1. Results of the Content Validity Index (CVI) assessment (relevance, clarity, appropriateness) for each item in the initial 23-item CD-Know version and whether each item was retained, removed or modified.

**Item number**	**Relevance**	**Clarity**	**Appropriateness**	**Included or modified**
1	0.82	*0.62*	*0.75*	Modified
2	0.83	0.84	0.87	Yes
3	*0.75*	*0.67*	*0.76*	No
4	0.80	*0.74*	*0.77*	Modified
5	0.93	0.90	0.87	Modified
6	0.98	0.92	0.94	Yes
7	0.83	0.79	*0.76*	No
8	0.84	0.79	0.78	Yes
9	0.88	0.78	0.83	Modified
10	0.95	0.91	0.95	Yes
11	0.92	0.89	0.93	Yes
12	0.90	0.83	0.90	Yes
13	0.82	0.81	0.82	Modified
14	0.90	0.80	0.85	Modified
15	0.85	*0.74*	0.82	Modified
16	0.91	0.82	0.87	Yes
17	0.94	0.93	0.90	Yes
18	0.85	0.86	0.86	No
19	0.89	0.86	0.89	Yes
20	0.89	0.83	0.87	Yes
21	0.87	0.84	0.88	Yes
22	*0.61*	*0.69*	*0.65*	No
23	0.79	*0.68*	0.78	No
