# Supplementary material for: Development and Validation of a Tool to Assess Disease-Related Knowledge in Children with Coeliac Disease
Source: J Clin Med. 2026 Jan 26;15(3):997. doi: 10.3390/jcm15030997 (PMC12898186; doi:10.3390/jcm15030997)
Supplement: Supplementary file 1 [file jcm-15-00997-s001.zip › jcm-4066389-Table S2.pdf]

Original versus revalidated CD-Know tool

FIRST VERSION	SECOND VERSION	THIRD VERSION	FINAL VERSION
**CD-Know pre- content validity**	**CD-Know Pilot & Validation 1**	**CD-Know Pilot 2**	**CD-Know Validation 2**
Which one of these describes coeliac disease?<br>a) An allergy<br>b) An immune response<br>c) A food sensitivity<br>d) Don't know	Which one of these <u>best</u> describes coeliac disease?<br>a) An allergy<br>b) An immune response<br>c) An intolerance<br>d) Don't know	Which of these <u>best</u> describes coeliac disease?<br>a) An enzyme deficiency<br>b) An immune response<br>c) An allergic response<br>d) Don't know	1. Which of these <u>best</u> describes coeliac disease?<br>a) An enzyme deficiency<br>b) An immune response<br>c) An allergic response<br>d) Don't know
Can coeliac disease be cured?<br>a) Yes<br>b) No<br>c) Don't know	Coeliac disease can be cured?<br>a) True<br>b) False<br>c) Don’t know	Which statement about coeliac disease is true?<br>a) You can grow out of it<br>b) It is lifelong<br>c) Medications can control symptoms<br>d) Don’t know	2. Which statement about coeliac disease is true?<br>a) You can grow out of it<br>b) It is lifelong<br>c) Medications can control symptoms<br>d) Don’t know
A gluten free diet may be low in which nutrients?<br>a) Fibre<br>b) Fat<br>c) Energy<br>d) Don't know	A gluten free diet may be low in:<br>a) Fibre<br>b) Fat<br>c) Calories<br>d) Don’t know	A gluten free diet may be low in:<br>a) Fibre<br>b) Fat<br>c) Calories<br>d) Don’t know	3. A gluten free diet may be low in:<br>a) Fibre<br>b) Fat<br>c) Calories<br>d) Don’t know
Wheat free is the same as gluten free<br>a) Yes<br>b) No<br>c) Don't know	Wheat free is the same as gluten free.<br>a) True<br>b) False<br>c) Don’t know	If a food states it is wheat free then it is always gluten free<br>a) True<br>b) False<br>c) Don’t know	4. If a food states it is wheat free then it is always gluten free<br>a) True<br>b) False<br>c) Don’t know
If a food label says 'starch' does this mean it always contains gluten?<br>a) Yes<br>b) No<br>c) Don't know	If a food label says ‘starch’ it always contains gluten?<br>a) True<br>b) False<br>c) Don’t know	If a food label says 'starch' it always contains gluten.<br>a) True<br>b) False<br>c) Don’t know	5. If a food label says 'starch' it always contains gluten.<br>a) True<br>b) False<br>c) Don’t know
What is gluten?<br>a) A protein<br>b) A food additive<br>c) A flavouring<br>d) Don't know	What is gluten?<br>a) A protein<br>b) A fat<br>c) A carbohydrate<br>d) Don’t know	What is gluten?<br>a) A protein<br>b) A fat<br>c) A carbohydrate<br>d) Don’t know	6. What is gluten?<br>a) A protein<br>b) A fat<br>c) A carbohydrate<br>d) Don’t know

Which grains can be eaten by people with coeliac disease? *(tick all that apply)*<br>☐ Buckwheat<br>☐ Wheat<br>☐ Spelt<br>☐ Barley<br>☐ Corn<br>☐ Rye<br>☐ Rice<br>☐ Quinoa	Which grains can be safely eaten by people with coeliac disease? (tick all that apply)<br>☐ Barley<br>☐ Buckwheat<br>☐ Rice<br>☐ Rye	Which grains can be safely eaten by people with coeliac disease? (select one option)<br>a) Buckwheat, rye, semolina<br>b) Polenta, quinoa, glutenous rice<br>c) Glutenous rice, cous-cous, buckwheat<br>d) Don't know	7. Which grains can be safely eaten by people with coeliac disease? (select one option)<br>a) Buckwheat, rye, semolina<br>b) Polenta, quinoa, glutenous rice<br>c) Glutenous rice, cous-cous, buckwheat<br>d) Don't know
If you are following a gluten-free diet and the menu states a dish may contain gluten, what should someone with coeliac disease do?<br>a) Choose another meal that is gluten free<br>b) Hope there is only a little gluten and order the dish<br>c) Remove the parts that contain gluten and eat<br>d) Don't know	If a restaurant menu states a dish may contain gluten, what should someone with coeliac disease do?<br>a) Choose another gluten free meal<br>b) Order and hope that there is no gluten<br>c) Remove the parts that may contain gluten<br>d) Don't know	You order a gluten free burger but it arrives with a regular bun. What should you do?<br>a) Ask for a new burger to be made<br>b) Ask to have the patty put in a gluten free bun<br>c) Just eat the patty<br>d) Don't know	8. You order a gluten free burger but it arrives with a regular bun. What should you do?<br>a) Ask for a new burger to be made<br>b) Ask to have the patty put in a gluten free bun<br>c) Just eat the patty<br>d) Don't know
What does the international symbol for gluten free look like?<br>a) A crossed wheat grain<br>b) A crossed slice of bread<br>c) A gluten traffic light<br>d) Don't know	What does the international symbol for gluten free look like?<br>a) A crossed grain<br>b) A crossed slice of bread<br>c) A gluten traffic light<br>d) Don't know	What is the international symbol for 'gluten free' look like?<br>a) A crossed grain<br>b) A crossed slice of bread<br>c) A gluten traffic light<br>d) Don't know	9. What is the international symbol for 'gluten free' look like?<br>a) A crossed grain<br>b) A crossed slice of bread<br>c) A gluten traffic light<br>d) Don't know
Herbal medicines and supplements are all gluten free<br>a) Yes<br>b) No<br>c) Don't know	All herbal medicines and supplements are all gluten free.<br>a) True<br>b) False<br>c) Don't know	All herbal medicines and supplements are gluten free.<br>a) True<br>b) False<br>c) Don't know	10. All herbal medicines and supplements are gluten free.<br>a) True<br>b) False<br>c) Don't know
		Coeliac disease may cause dental enamel problems<br>a) True<br>b) False<br>c) Don't know	11. Coeliac disease may cause tooth enamel problems<br>a) True<br>b) False<br>c) Don't know

		Which part of the gut is affected by coeliac disease?<br>a) Stomach<br>b) Small bowel<br>c) Large bowel<br>d) Don't know	12. Which part of the gut is affected by coeliac disease?<br>a) Stomach<br>b) Small bowel<br>c) Large bowel<br>d) Don't know
Are some people more likely to have coeliac disease?<br>a) Yes<br>b) No<br>c) Don't know	Coeliac disease is a genetic condition:<br>a) Yes<br>b) No<br>c) Don't know	If I have coeliac disease, my parents and siblings are:<br>a) More likely to have coeliac disease<br>b) Less likely to have coeliac disease<br>c) The same as everyone else<br>d) Don't know	13. If I have coeliac disease, my parents and siblings are:<br>a) More likely to have coeliac disease<br>b) Less likely to have coeliac disease<br>c) The same as everyone else<br>d) Don't know
		Can someone with coeliac disease have gluten 6 months after diagnosis if they have no symptoms?<br>a) Yes<br>b) No<br>c) Don't know	14. Can someone with coeliac disease have gluten 6 months after diagnosis if they have no symptoms?<br>a) Yes<br>b) No<br>c) Don't know
What kitchen items are NOT safe for people with coeliac disease to share? *(tick all that apply)*<br>☐ Microwave<br>☐ Toaster<br>☐ Washing up liquid<br>☐ Cooking oil<br>☐ Jam/spreads<br>☐ Fridge	What kitchen items are NOT safe for people with coeliac disease to share with people who eat gluten? (Tick all that apply)<br>☐ The same microwave<br>☐ The same serving spoon<br>☐ Washing up liquid<br>☐ Shared knife in jam/spreads	Gluten free food cooked on a barbecue/grill is always safe:<br>a) Yes<br>b) No<br>c) Don't know	15. Gluten free food cooked on a barbecue/grill is always safe:<br>a) Yes<br>b) No<br>c) Don't know
At what age do people find out they have coeliac disease?<br>a) As a child<br>b) As an adult<br>c) At any age<br>d) Don't know	When might people find out they have coeliac disease?<br>a) As a child<br>b) As an adult<br>c) At any age<br>d) Don't know		

How is coeliac disease treated?<br>a) Drugs<br>b) Diet<br>c) Surgery<br>d) Don't know	How is coeliac disease currently treated?<br>a) Medication<br>b) Diet<br>c) Surgery<br>d) Don't know		
What diet should people with coeliac disease follow?<br>a) Lactose free<br>b) No special diet<br>c) Gluten-free<br>d) Don't know	What diet should people with coeliac disease follow?<br>a) Lactose free diet<br>b) Low carbohydrate diet<br>c) Gluten free diet<br>d) Don't know		
Some naturally gluten free foods may have gluten added to them<br>a) Yes<br>b) No<br>c) Don't know	Some naturally gluten free foods may have gluten added to them.<br>a) True<br>b) False<br>c) Don't know		
All medicines are gluten free<br>a) Yes<br>b) No<br>c) Don't know	Which statement about medicines is true<br>a) All medicines are gluten free<br>b) Most medicines are gluten free but should always be checked<br>c) All tablets contain gluten so a liquid version should be requested<br>d) Don't know		
How strict should the diet be for someone with coeliac disease?<br>a) It depends on the person<br>b) A strict gluten free diet all the time<br>c) Only a strict gluten free diet when they have symptoms<br>d) Don't know	How strict should the diet be for someone with coeliac disease?<br>a) It depends on the person<br>b) A strict gluten free diet all the time<br>c) Only strict gluten free when they have symptoms<br>d) Don't know		
How do you decide if a packet of potato chips contains gluten?<br>a) Read the ingredients list<br>b) Assume they are gluten free<br>c) Only eat organic potato chips<br>d) Don't know	How do you decide if a packet of potato chips contains gluten?<br>a) Assume they are gluten free as have eaten them before<br>b) Only eat organic potato chips<br>c) Read the ingredients list		

	d) Don’t know		
Do you need to check hair and beauty products for gluten?<br>a) Yes<br>b) No<br>c) Don't know			
Do doctors know the cause of coeliac disease?<br>a) Yes<br>b) No<br>c) Don't know			
People with coeliac disease can be just as healthy as those without it<br>a) Yes<br>b) No<br>c) Don't know			
What are common symptoms of untreated coeliac disease?			
